# When Naked Became Armored: An Eight-Gene Phylogeny Reveals Monophyletic Origin of Theca in Dinoflagellates

**DOI:** 10.1371/journal.pone.0050004

**Published:** 2012-11-19

**Authors:** Russell J. S. Orr, Shauna A. Murray, Anke Stüken, Lesley Rhodes, Kjetill S. Jakobsen

**Affiliations:** 1 Microbial Evolution Research Group (MERG), Department of Biology, University of Oslo, Oslo, Norway; 2 Ecology and Evolution Research Centre and School of Biotechnology and Biomolecular Sciences, University of New South Wales, Sydney, Australia; 3 Sydney Institute of Marine Sciences, Mosman, New South Wales, Australia; 4 Cawthron Institute, Nelson, New Zealand; 5 Centre for Ecological and Evolutionary Synthesis (CEES), Department of Biology, University of Oslo, Oslo, Norway; University of Connecticut, United States of America

## Abstract

The dinoflagellates are a diverse lineage of microbial eukaryotes. Dinoflagellate monophyly and their position within the group Alveolata are well established. However, phylogenetic relationships between dinoflagellate orders remain unresolved. To date, only a limited number of dinoflagellate studies have used a broad taxon sample with more than two concatenated markers. This lack of resolution makes it difficult to determine the evolution of major phenotypic characters such as morphological features or toxin production e.g. saxitoxin. Here we present an improved dinoflagellate phylogeny, based on eight genes, with the broadest taxon sampling to date. Fifty-five sequences for eight phylogenetic markers from nuclear and mitochondrial regions were amplified from 13 species, four orders, and concatenated phylogenetic inferences were conducted with orthologous sequences. Phylogenetic resolution is increased with addition of support for the deepest branches, though can be improved yet further. We show for the first time that the characteristic dinoflagellate thecal plates, cellulosic material that is present within the sub-cuticular alveoli, appears to have had a single origin. In addition, the monophyly of most dinoflagellate orders is confirmed: the Dinophysiales, the Gonyaulacales, the Prorocentrales, the Suessiales, and the Syndiniales. Our improved phylogeny, along with results of PCR to detect the *sxtA* gene in various lineages, allows us to suggest that this gene was probably acquired separately in *Gymnodinium* and the common ancestor of *Alexandrium* and *Pyrodinium* and subsequently lost in some descendent species of *Alexandrium*.

## Introduction

Approximately 2000 species of living dinoflagellates are known, most of which are found in marine habitats [1]. Species vary widely, in characteristics such as cell morphology and modes of nutrition (e.g., autotrophy, heterotrophy, mixotrophy, symbiosis, and parasitism) [1], [2], [3]. Dinoflagellate taxonomy is based on morphological characters such as the presence of a dinokaryon, and the arrangement and shape of thecal plate-containing amphiesmal vesicles. A dinokaryon, a modified nucleus containing permanently condensed fibrillar chromosomes [1], [4], [5], [6], [7], [8], is present in the “core” dinoflagellates, but lacking from the “pre-dinoflagellate” lineages Oxyrrhinaceae and the Syndiniales [3], [9]. The Blastodiniales and Noctilucales lack a dinokaryon during particular life cycle stages. For this reason, it has been hypothesized that these lineages are basal [6], [10], [11], [12], although recent evidence suggests that the Blastodiniales may have diverged more recently [13], [14], [15].

The arrangement of the thecal plate bearing amphiesmal vesicles is an important character in distinguishing clades of dinoflagellates [16]. The thecate (armored) orders (Dinophysiales, Gonyaulacales, Peridiniales, Prorocentrales and Suessiales) have comparatively fewer, large amphiesmal vesicles in distinctive patterns, with cellulosic material in the vesicles. Athecate (unarmored or naked) taxa, however (Gymnodiniales, Noctilucales and Syndiniales) often contain hundreds of alveoli lacking cellulosic material, and therefore relationships are determined based on other features, such as the presence and shape of grooves on the cell surface or on the cell apex, and the shape of the epicone [17], [18], [19], [20].

The monophyly of dinoflagellates and their sister relationships to the Apicomplexa have been established from previous dinoflagellate phylogenies, as well as global eukaryotic phylogenies [16], [21], [22], [23], [24], [25], [26]. Fossil evidence suggests that these groups diverged earlier than 400 Ma [27], and that the species *Alexandrium tamarense* is a fairly recent dinoflagellate lineage, emerging between 23–45 Ma [28]. The phylogenetic relationship between the dinoflagellate orders, however, is unresolved, with a lack of statistical support for the phylogenetic backbone [5], [16], [23], [24], [29], [30].

Early molecular phylogenetic studies of dinoflagellate relationships were based on ribosomal rDNA, either partial large-subunit (LSU) [5], [17], [31], or most frequently, small-subunit (SSU) [5], [24], [32], [33]. However, the low proportion of informative characters resulted in poor resolution despite broad taxon sampling [5], [24], [33]. Concatenated rDNA inferences have added more resolution, proving useful in the interpretation of genus level relationships [5], [29], [30], [34], [35]. However, inter-order relationships remain unclear, with deep branches receiving little or no statistical support, making trends difficult to infer [5], [16], [36]. Recently, the use of protein genes for phylogenetic inference of dinoflagellates has increased, in particular actin, alpha- and beta-tubulin [22], *hsp90* [16], [24], and the mitochondrial cytochrome genes [23]. However, as yet few have inferred a broad dinoflagellate phylogeny based on more than two concatenated genetic markers [16], [23]. Presently, sequence data is only available for approximately 10% or less of the known dinoflagellate species diversity [29]. The identification of the marine alveolate lineages (MALV), gives an insight into the large parasitic Syndiniales diversity [37], [38], [39], [40], [41], [42]. A bias toward the photosynthetic taxa also exists, as a large proportion of heterotrophic species, which make up approximately 50% of the true dinoflagellate lineage [43], are difficult or impossible to culture.

A well-resolved dinoflagellate phylogeny is essential to understanding the evolution of toxin synthesis in this phylum. Approximately 100 known species of dinoflagellates produce a variety of toxins, that can accumulate in the water column as Harmful Algal Blooms (HABs) [44]. Saxitoxin (STX), and its analogues, is one such toxin that can cause severe symptoms upon consumption of vector species [45], [46]. STX is synthesized by eukaryotic marine dinoflagellates and freshwater cyanobacteria [47], [48]. The toxins appear to be synthesized by similar processes in cyanobacteria and dinoflagellates [49]. The biosynthetic pathway and genes responsible for STX- synthesis are known from cyanobacterial species [50], [51], [52], [53], [54]. The genes common between these clusters have been defined as “core” genes [46], [55]. One such core gene, *sxtA*, the unique starting gene of STX synthesis, has recently been identified in the dinoflagellates *Gymnodinium catenatum* and multiple species within the genus *Alexandrium* [56]. The origin of this gene cluster within the dinoflagellates may have occurred by way of a horizontal gene transfer (HGT) event between an ancestral STX-producing bacterium and the dinoflagellates, before *Alexandrium* and *Pyrodinium* diverged [56]. Thus the ability to produce STX may have been secondarily lost for some descendent species. As the *sxtA* sequence of *Gymnodinium catenatum,* in the order Gymnodiniales, branches within *Alexandrium,* an independent acquisition of STX from a dinoflagellate-dinoflagellate transfer has been postulated [56]. As the phylogenetic relationship between dinoflagellate species remains unresolved, trends in the evolution of the genetic basis for the synthesis of STX or other toxins cannot be established [23].

The aim of this study is to improve the resolution of the dinoflagellate phylogeny by sampling a broad range of both taxa and genes using concatenated alignments. This will allow us to address relationships between orders and identify possible phylogenetic trends in the evolution of STX production and other major phenotypic characters, such as morphological traits. To achieve this, 55 sequences for eight molecular markers were amplified from 13 species, spanning four orders. A concatenated phylogenetic approach was used with all orthologous database sequences. Furthermore, we tested 20 species from five orders for presence of *sxtA1* and *sxtA4.*

## Methods

Our use of order, family and genus-level names follows the taxonomic revision of Fensome et al. (1993), and its recent update Fensome et al. (2008), made publically available online (http://dinoflaj.smu.ca/) [6], [57]. However, the following amendments are used: “core” dinoflagellates for dinokaryota [58], family Kareniaceae [17], [59], the inclusion of *Brachidinium* in Kareniaceae [60], genus *Thoracosphaera* [61], genus *Adenoides* [62], *Biecheleria baltica* (syn. *Woloszynskia halophila*) [63], *Pelagodinium beii* (syn. *Gymnodinium beii*) [64], and *Protodinium simplex* (syn. *Gymnodinum* simplex) [58].

### Culturing

The dinoflagellate strains used in this study (Table 1) were grown in L1 media [65] or GSe media [66] at 16–25°C. In addition, *Polarella glacialis* CCMP2088 was grown at 5°C, all with a 12∶12 h light-dark photoperiod and a photon irradiance of ∼100 mmol photons m^−2^ s^−1^. Strains were not maintained axenically. The identity of each strain was confirmed by amplifying the 18S rDNA gene using the primer pairs NSF83 - 1528R and 18sF8 - ITSR01 [67], [68], [69]. Inclusion of *Pyrodinium bahamense* in the study would be desirable as it produces saxitoxin, however no culture was available to us.

**Table 1:** List of dinoflagellate strains used in this study and whether *sxtA1* and *sxtA4* were PCR amplified.

Species/Taxon	Strain	*sxtA1*	*sxtA4*
*Adenoides eludens*	CCMP1891	n.d.	n.d.
*Alexandrium fundyense*	CCMP1719	+ [56]	+ [56]
*Alexandrium minutum*	CCMP113	+ [56]	+ [56]
*Amphidinium carterae*	UIO081	n.d.	n.d.
*Amphidinium massartii*	CS-259	n.d. [56]	n.d. [56]
*Amphidinium mootonorum*	CAWD161	n.d.	n.d.
*Azadinium spinosum*	RCC2538	n.d.	n.d.
*Ceratium longipes*	CCMP1770	n.d.	n.d.
*Coolia monotis*	CAWD98	n.d.	n.d.
*Gambierdiscus australes*	CAWD148	n.d.	n.d.
*Gymnodinium aureolum*	SCCAP K-1561	n.d.	n.d.
*Heterocapsa triquetra*	RCC2540	n.d.	n.d.
*Karlodinium veneficum*	RCC2539	n.d.	n.d.
*Lepidodinium chlorophorum*	RCC2537	n.d.	n.d.
*Lingulodinium polyedrum*	CCMP1931	n.d.	n.d.
*Pentapharsodinium dalei*	SCCAP K-1100	n.d.	n.d.
*Polarella glacialis*	CCMP2088	n.d.	n.d.
*Prorocentrum micans*	UIO292	n.d.	n.d.
*Prorocentrum minimum*	UIO085	n.d.	n.d.
*Protoceratium reticulatum*	CAWD99	n.d.	n.d.
*Pyrocystis noctiluca*	CCMP732	n.d.	n.d.
*Scrippsiella trochoideae*	BS-46	n.d.	n.d.
*Thecadinium kofoidii*	SCCAP K-1504	n.d.	n.d.

n.d. not detected. +amplified sequence.

### DNA and RNA Isolation, cDNA Synthesis, PCR Amplification, Sequencing and Assembly

Genomic DNA and total RNA were isolated from 20 ml cultures in the exponential growth phase, centrifuged for 2 min at 12,000×*g*, washed with PBS and bead-beaten on dry ice with the FastPrep-24 from Medinor (20 s, speed 4) using 1.4 mm beads (Medinor). For DNA the CTAB method [70] or Invitrogen ChargeSwitch gDNA plant kit (Invitrogen) were utilized. For total RNA the Invitrogen ChargeSwitch TotalRNA cell kit (Invitrogen) or RNeasy Plant Mini kit (Qiagen) were used in accordance with supplied protocol. First strand cDNA was synthesized with Invitrogen 3′ RACE system (Invitrogen) following the high GC protocol and utilizing the (AP) adapter primer, or with Invitrogen Superscript First-Strand Synthesis system (Invitrogen). DNA, RNA and cDNA quality was checked with a NanoDrop spectrophotometer (ThermoScientific).

The genes amplified in this study 18S rDNA (Small subunit), 5.8S rDNA, 28S rDNA (Large subunit), actin, beta-tubulin, cytochrome *b* (*cob*), cytochrome c oxidase subunit I (*cox1*), and heat-shock protein 90 (*hsp90*), were determined from dinoflagellate sequence availability within NCBI. Mixing of gDNA and cDNA sequences for phylogenetic inference may produce invalid results due to widespread mRNA editing in dinoflagellates [23]. Thus, mRNA was utilized for *cob* and *cox1,* as only a single functional sequence is reported, in comparison to multiple genomic copies [23]. Likewise, actin in dinoflagellates is present in a variable number of copies in the genome, including pseudogenes; therefore mRNA was again favored [71]. Beta-tubulin (mRNA) and *hsp90* (gDNA) were determined from sequence availability. Template was only PCR amplified for the genes and strains lacking Genbank sequence data. cDNA/gDNA template was PCR amplified using Qiagen HotStarTaq *Plus* polymerase (Qiagen), Bioline Mytag polymerase (Bioline) or BD Advantage 2 polymerase (Clonetech) in the presence of 10% BSA in a MJ Research PTC-200 Thermo Cycler (MJ Research) with the following PCR conditions: an initial denaturing before 35 cycles of (1) 30 sec denaturing, (2) 30 sec annealing (variable temperature, see Table S1 for T_M_), and (3) 1–2 minute extension, with a final 10 minute extension at the same temperature. PCR products were gel excised using Promega Wizard SV Gel and PCR Clean-Up System (Promega), before direct sequencing with an ABI3730 DNA analyzer (Applied Biosystems) using combinations of primers (Table 2; Table S1). The universal primers used in this study have been designed using Primaclade based on alignments constructed from multiple orthologous dinoflagellate sequences. [72]. Melting temperature (T_M_) was calculated using OligoCalc [73]. Sequences were quality checked and assembled using the Phred/Phrap/Consed [74] package under default settings. Additional manual editing was performed in MacCladev4.07 [75]. The presence of *sxtA*1 and *sxtA*4 genes were tested for all dinoflagellate strains following the protocol described in [56]. The *sxtA*1 fragment was amplified with primers sxt001 & sxt002 (∼550 bp) and the *sxtA*4 fragment with the primers sxt007 & sxt008 (∼750 bp) (Table S1). A positive gDNA control from *A. fundyense* CCMP1719 or *A. minutum* CCMP113 was utilized in all *sxtA* PCRs.

**Table 2:** Primers designed specifically for this study: Annealing site is an approximation and can vary slightly between species; *Prorocentrum minimum* was used as a reference.

Primer name	Primer direction	Primer sequence 5′-3′	Annealing site 5′-3′
DinoActinF1	F	GAYGARGCDCAGAGCAAGC	169–187
DinoActinF2	F	ATCATGGTSGGCATGGAC	130–147
DinoActinR2	R	TTGGAGATCCACATCTGCTG	1060–1079
Actin943F	F	ATGAAGATCAAGGTNGTNGC	976–995
DinoBtubF1	F	GGHGCNAARTTYTGGGAGG	49–67
DinoBtubF2	F	GDGCMAAGTTCTGGGARGT	50–68
DinoBtubR1	R	AGGTGGTTCAGGTCHCCGTA	665–683
DinoBtubR2	R	YTCWCCDGTGTACCARTGCAA	1183–1203
Btub305F	F	TSCAGGGBTTCCAGATGT	389–406
Btub305R	R	ACATCTGGAAVCCCTGSA	389–406
DinoCYTbF1	F	WCHGGWATCTTCTTAGCTTTACATTA	73–98
DinoCYTbF2	F	TTRTCACWGGAATCTTMTTAGSTTT	68–92
CYTB343F	F	GGACAAATGAGTTTMTGGGG	343–362
DinoCOXF2	F	CCATTAAGCACKTCTTTYMTGAGTT	349–373
COX211F	F	ATCTTTCAAGGRTCTCCWGAAGTG	211–234
COX631F	F	TTTGGAGGAGATCCTRTWCTCTAT	631–654
COX1021R	R	CCAAGAATTACTCCTGTTGASCC	1021–1043
DinoRhsp90F2	F	ATCCGSTAYGAGTCVATCAC	46–65
DinoRhsp90R2	R	ACCTTGTCKCCSARVACCT	1577–1595

For a full list of primers, primer pairs and annealing temperatures used see Table. S1.

### Phylogenetic Inferences

All sequences generated in this study as well as dinoflagellate orthologous sequences in the NCBInr nucleotide and EST databases http://www.ncbi.nlm.nih.gov/(as of 12.2011) for each gene were separated into their respective datasets. The three-rDNA genes (18S, 5.8S, and 28S) were separately aligned using the MAFFTv6 Q-INS-I model [76], [77], [78], considering secondary RNA structure (default parameters used). The five protein coding datasets (actin mRNA, beta-tubulin mRNA, *cob* mRNA, *cox1* mRNA, and *hsp90* gDNA) were separately aligned at the nucleotide level based on the corresponding amino acid alignment, as to maintain codon integrity, inferred with MAFFTv6 G-INS-I model (default parameters used). To increase phylogenetic signal, allowing for synonymous substitutions, the nucleotide sequence (3^rd^ codon removed) was used for subsequent inferences. Outgroup taxa (Apicomplexa) was established from previous dinoflagellate phylogenies [16], [23], [24], as well as global eukaryotic phylogenies that concur in placing this as the closest extant relative to the dinoflagellates [25], [26]. In-group taxa (dinoflagellata) required both 18S and 28S rDNA sequence data, thus *Blastodinium*, having only 18S rDNA was excluded. The only exception was *Ceratocorys horrida*; this species’ 28S rDNA sequence is not available, though as it had available cytochrome sequences, and as the only representative from this family, its inclusion was considered important. The resulting single gene alignments were subsequently checked manually using MacCladev4.07 [75]. The eight separate alignments were then checked with Gblocks v0.91b [79], under the least stringent parameters (small final block, gap positions in final block and less strict flanking), to exclude poorly aligned positions and divergent regions from subsequent phylogenetic inferences. The alignments were then concatenated into the following supermatrices; (1) rDNA; (18S+5.8S+28S), (2) rDNA+nuclear protein; (18S+5.8S+28S+actin+beta-tubulin+*hsp90*) and (3) rDNA+mitochondrial+nuclear protein; (18S+5.8S+28S+*cob*+*cox1*+actin+beta-tubulin+*hsp90*), a reduced dataset was additionally constructed from the previous two, excluding taxa with only rDNA signal (lacking protein coding gene data); done to evaluate effects of missing characters and taxon sampling on the inferences.

The supermatrix (concatenated) approach provides support not always apparent with fewer genes [80]. To reduce missing data and improve phylogenetic placement, concatenated *Hematodinium* sequences were a composite (*in silico* chimeric) of closely related intra-genus species (*H. perezi*, *Hematodinium* sp., *Hematodinium* sp. ex *Callinectes sapidus*, and *Hematodinium* sp. ex *Nephrops norvegicus*; Table S2).

Taxa have not been excluded from the inferred supermatrices with “missing characters” as a criteria, as phylogenetic estimates including incomplete taxa show little evidence to support taxa exclusion based on missing data [81]. Addition of incomplete taxa, even <10% complete, can be equally beneficial to a phylogeny as 100% complete taxa, improving resolution at the genus level, placing with strong statistical support, and even subdividing misleading long branches [80], [81], [82], [83]. The critical factor for taxa placement is not character absence, but the quality and number of those present [81]. All concatenated datasets were then analyzed with MODELTEST [84] to establish the optimal model of nucleotide evolution; for all alignments the (General Time Reversible) GTR model was preferred for both the Akaike and Bayesian information Criterion (AiC and BiC). Maximum Likelihood (ML) analyses were performed with RAxML-VI-HPCv7.2.6, GTRCAT model with 25 rate categories [85], [86]. The most likely topology was established from 100 separate searches and bootstrap analyses were performed with 500 pseudoreplicates. Bayesian analyses were carried out with MrBayes MPI version 3.1.2 [87], [88]. Trees were generated from two independent runs with one heated and one cold chain in the Markov Chain Monte Carlo (MCMC) with 40,000,000 generations, sampling every 1000. Analyses ran until the average standard deviation of split frequencies were 0.01. Burn-in trees were set based on the assessment of likelihood plots and convergence diagnostics implemented in MrBayes. The Potential Scale Reduction Factor (PSRF) values for all inferences were ∼1.0, indicating a good posterior probability distribution sample. The majority rule tree and posterior probabilities for each inference was constructed from a consensus of the sampled post burn-in trees. Topological congruence between the inferred phylogenies were calculated using the *l_cong_* index: http://max2.ese.u-psud.fr/bases/upresa/pages/devienne/index.html [89].

*Noctiluca scintillans* was excluded from the presented concatenated analyses as its cryptic and inconsistent placement reduced phylogenetic support. However, its “most probable” placement was determined from parallel Bayesian inferences.

Previous phylogenies based on the mitochondrial cytochrome genes, *cob* and *cox1*, place *Heterocapsa* basal within the dinoflagellate lineage [23], [90], a possible phylogenetic artifact as a result of a faster mutation rate [23]. As this position is inconsistent with morphological data and phylogenies without mitochondrial genes [16], [30], we investigated this further. A simple distance-based comparative rate test was used to measure the divergence of the different genes for *Heterocapsa triquetra* to that of the ingroup (“core” dinoflagellates) [91], [92]. In this context, the comparative rate was defined as the ratio of the pairwise distances of *Heterocapsa* to the ingroup taxa, compared with the mean distance between the same ingroup taxa. Here we considered *A. carterae*, *A. eludens*, *A. minutum*, *A. spinosum*, *G. aureolum*, *K. veneficum*, *P. glacialis*, *P. minimum*, and *S. trochoideae* to form the ingroup. The distance from *Heterocapsa* to the ingroup taxa was divided by the mean distance of the ingroup taxa to each other. The pairwise distances between all taxa were calculated using RAxML [85], [86] with the -x option and GTRGAMMA model for each individual gene alignment as well as the rDNA, nuclear protein and cytochrome concatenated alignments. Subsequently, *cob* and *cox1* solely for *Heterocapsa* were excluded from inferences.

All model estimation and phylogenetic analyses were done on the freely available Bioportal [93] at the University of Oslo (http://www.bioportal.uio.no/).

## Results

### Sequence Amplification and Assembly

Fifty-five dinoflagellate sequences from 13 species and four orders were successfully amplified for 18S, 5.8S, 28S, actin, beta-tubulin, *cob*, *cox1*, and *hsp90.* All sequences generated in this study have been deposited in Genbank under the accession numbers, 18S: JX262491 and JX262492, 5.8S: JX262493-JX262497, 28S: JX262498, actin: JX262499-JX262509, beta-tubulin: JX262510-JX262519, *cox1*: JX262520-JX262529, *cob*: JX262530-JX262538, and *hsp90*: JX262539-JX262545.

Alignments generated in this study represent the broadest taxon sampling and character number inferred for the dinoflagellates to date. They have been made freely available at TreeBASE (http://www.treebase.org/treebase-web/home.html) under the accession URL: http://purl.org/phylo/treebase/phylows/study/TB2:S12493.

*Amphidinium carterae* (UIO081), *Ceratium longipes* (CCMP1770), *Coolia monotis* CAWD98, *Heterocapsa triquetra* (RCC2540), *Karlodinium veneficum* (RCC2539), *Lingulodinium polyedrum* (CCMP1931), *Prorocentrum micans* (UIO292), *Prorocentrum minimum* (UIO085), *Pyrocystis noctiluca* (CCMP732) and *Protoceratium reticulatum* CAWD99 were only used for *sxtA* detection via PCR.

### The Phylogeny of Dinoflagellates

**Figure 1:**
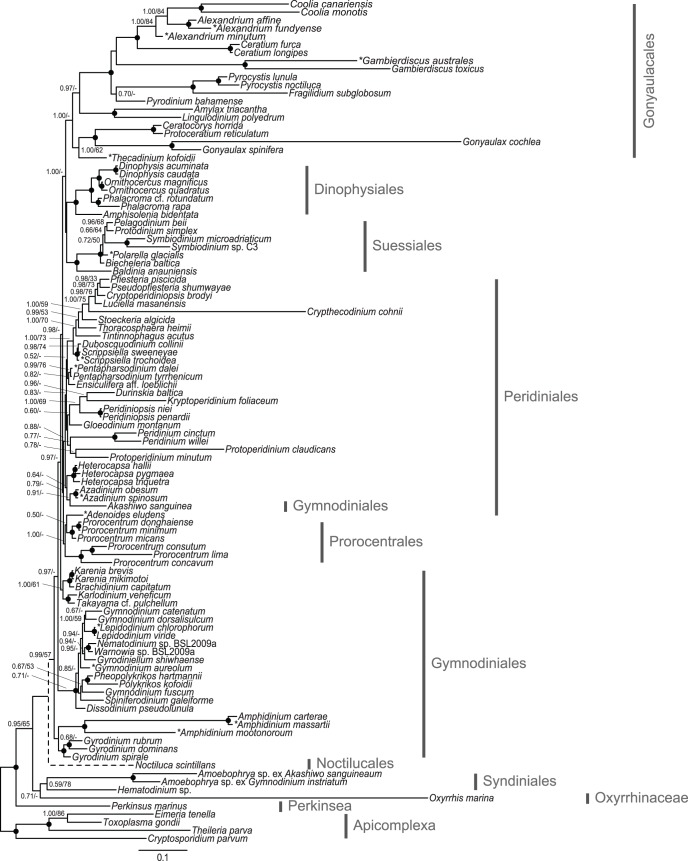
Phylogenetic tree of dinoflagellates inferred from rDNA. Concatenated phylogeny, inferred from 18S+5.8S+28S rDNA (2900 characters). The tree is reconstructed with Bayesian inference (MrBayes). Numbers on the internal nodes represent posterior probability and bootstrap values (>50%) for MrBayes and RAxML (ordered; MrBayes/RAxML). Black circles indicate a posterior probability value of 1.00 and bootstrap >90%. *N. scintilans* is represented with a dashed branch as this taxon was excluded from the inference; alternatively its most “probable” placement was determined from a parallel Bayesian analysis. * Denotes taxa sequences generated from this study. See Table S2 for a full listing of accessions used.

**Figure 2:**
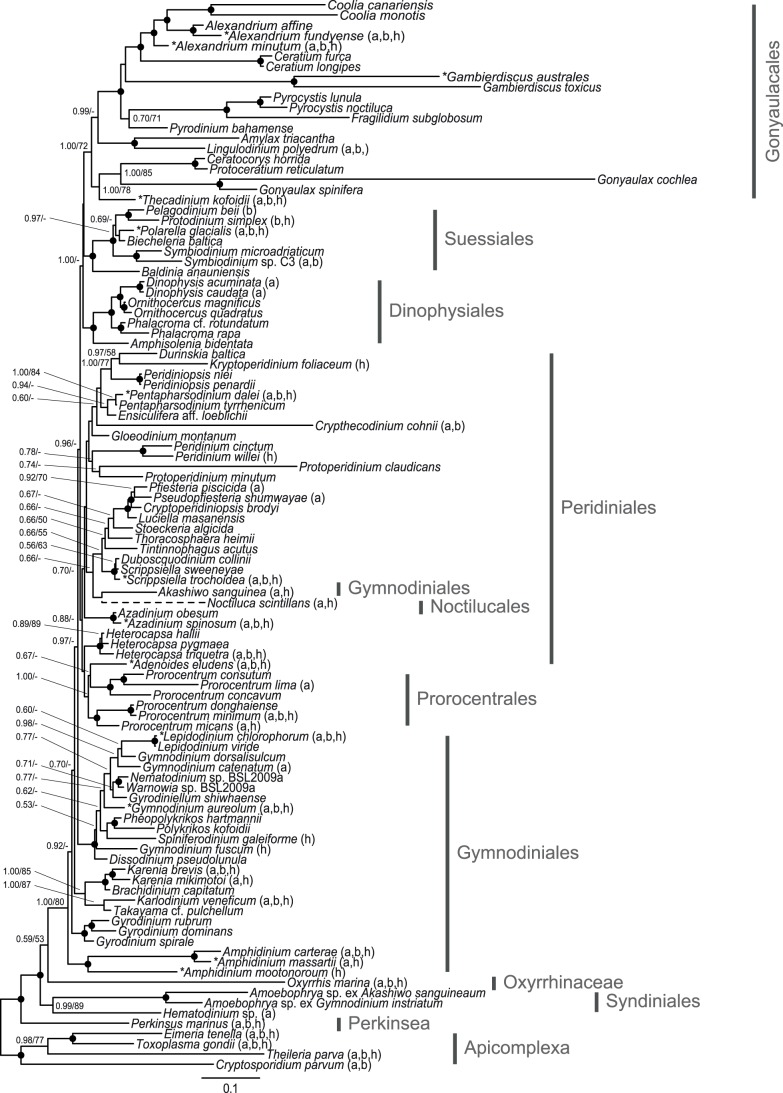
Phylogenetic tree of dinoflagellates inferred from rDNA and nuclear protein genes. Concatenated phylogeny, inferred from 18S+5.8S+28S+actin+beta-tubulin+*hsp90* (5626 characters). The tree is reconstructed with Bayesian inference (MrBayes). Numbers on the internal nodes represent posterior probability and bootstrap values (>50%) for MrBayes and RAxML (ordered; MrBayes/RAxML). Black circles indicate a posterior probability value of 1.00 and bootstrap >90%. *N. scintilans* is represented with a dashed branch as this taxon was excluded from the inference; alternatively its most “probable” placement was determined from a parallel Bayesian analysis. * Denotes taxa sequences generated from this study. See Table S2 for a full listing of accessions used. Non-ribosomal gene presence for each taxon is represented in brackets behind each species name (a: actin, b: beta-tubulin, h: *hsp90*).

**Figure 3:**
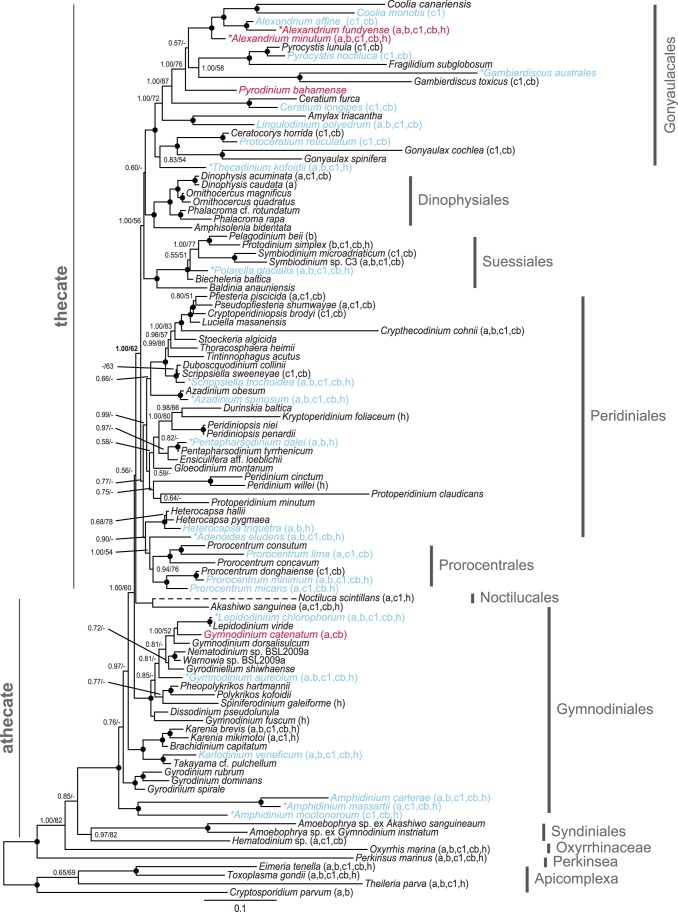
Phylogenetic tree of dinoflagellates inferred from rDNA, mitochondrial and nuclear protein genes. Concatenated phylogeny, inferred from 18S+5.8S+28S+*cob*+*cox1*+actin+beta-tubulin+*hsp90* (7138 characters). The tree is reconstructed with Bayesian inference (MrBayes). Numbers on the internal nodes represent posterior probability and bootstrap values (>50%) for MrBayes and RAxML (ordered; MrBayes/RAxML). Black circles indicate a posterior probability value of 1.00 and bootstrap >90%. *N. scintilans* is represented with a dashed branch as this taxon was excluded from the inference; alternatively its most “probable” placement was determined from a parallel Bayesian analysis. The cytochrome genes *cob* and *cox1* for *H. triquetra* were excluded from the inference, a parallel phylogeny including these genes for this taxon can be seen in Figure S1. * Denotes taxa sequences generated from this study. See Table S2 for a full listing of accessions used. Red font indicates *sxtA* presence and blue font indicates no *sxtA* detection. Non-ribosomal gene presence for each taxon is represented in brackets behind each species name (a: actin, b: beta-tubulin, c1: *cox1*, cb: *cob*, h: *hsp90*). The phylogenetic support for the thecate/athecate split is highlighted with bold type.

**Figure 4:**
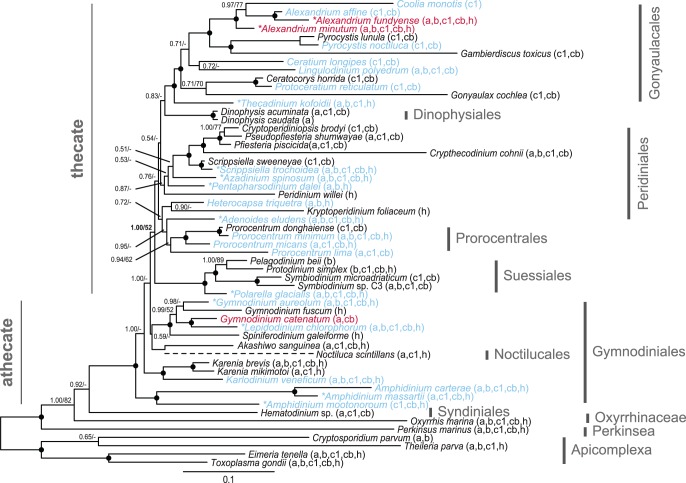
Phylogenetic tree of dinoflagellates inferred from rDNA, mitochondrial and nuclear protein genes (reduced phylogeny). Concatenated phylogeny, inferred from 18S+5.8S+28S+*cob*+*cox1*+actin+beta-tubulin+*hsp90* (7138 characters). This phylogeny was inferred excluding taxa with only rDNA signal; done to evaluate effects of missing characters and taxon sampling on the inference shown in Fig. 3. The tree is reconstructed with Bayesian inference (MrBayes). Numbers on the internal nodes represent posterior probability and bootstrap values (>50%) for MrBayes and RAxML (ordered; MrBayes/RAxML). Black circles indicate a posterior probability value of 1.00 and bootstrap >90%. *N. scintilans* is represented with a dashed branch as this taxon was excluded from the inference; alternatively its most “probable” placement was determined from a parallel Bayesian analysis. The cytochrome genes *cob* and *cox1* for *H. triquetra* were excluded from the inference. * Denotes taxa sequences generated from this study. See Table S2 for a full listing of accessions used. Red font indicates *sxtA* presence and blue font indicates no *sxtA* detection. Non-ribosomal gene presence for each taxon is represented in brackets behind each species name (a: actin, b: beta-tubulin, c1: *cox1*, cb: *cob*, h: *hsp90*). The phylogenetic support for the thecate/athecate split is highlighted with bold type.

All inferred dinoflagellate phylogenies show good topological congruence with an *l_cong_ P*-value <0.05 (Fig. 1, 2, 3, 4). Also the comparison of topologies inferred from separate rDNA and protein coding gene datasets demonstrated good congruence (Fig. S2). Removal of long-branching taxa had minimal topological impact (data not shown). In addition, the inference of the corresponding translated supermatrices had minimal topological impact (data not shown). Resolution was limited for the eight single gene phylogenies (Fig. S4, S5, S6, S7, S8, S9, S10, S11).

For interpretation of the phylogenetic inferences (Fig. 1, 2, 3, 4; Table 3), statistical support is defined as: full 1.00PP/100BP, high >90BP, moderate >65BP, and low >50BP. Dinoflagellate in-group monophyly was inferred for all datasets with support varying from moderate, to high (Fig. 2, 3, 4; Table 3). The first, and most basal, dinoflagellate order to diverge from the main branch was the Oxyrrhinaceae (Fig. 3–4). This position was unsupported, with Syndiniales being alternatively recovered as the most basal in the rDNA+nuclear protein dataset (Fig. 2). Both orders formed a sister relationship in the rDNA inference (Fig. 1). The Syndiniales clade was constantly recovered with moderate support (Fig. 1, 2, 3; Table 3). The “pre-dinoflagellate” Oxyrrhinaceae and Syndiniales lineages were excluded from the “core” dinoflagellates with support varying from low to full (Fig. 1, 2, 3, 4, Table 3).

**Table 3:** Comparison of the phylogenetic support (Posterior probability and bootstrap) received for the dinoflagellate orders, major lineages, clades and nodes in the inferences of Fig. 1, 2, 3, 4 (ordered; MrBayes/RAxML).

Figure	1	2	3	4
Dataset	rDNA	rDNA+nuclear protein	rDNA+mitochondrial+nuclear protein	Reduced rDNA+mitochondrial+nuclear protein
**Taxa/Characters**	**104/2900**	**104/5626**	**104/7138**	**56/7138**
**Order/Lineage/clade**				
Dinoflagellates	0.95/65	1.00/95	1.00/82	1.00/82
Syndiniales	0.59/78	0.99/89	0.97/82	–
“Core” dinoflagellates	0.99/57	1.00/80	1.00/98	1.00/100
Gymnodiniales	(polyphyletic)	(polyphyletic)	(paraphyletic)	(paraphyletic)
*Amphidinium*	1.00/91	1.00/91	1.00/97	1.00/92
*Gyrodinium*	1.00/94	1.00/100	1.00/98	–
Kareniaceae	1.00/61	1.00/85	1.00/92	1.00/100
*Gymnodinium sensu stricto*	1.00/97	1.00/100	1.00/100	1.00/100
Thecate/athecate split	–	–	1.00/62	1.00/52
Prorocentrales	(paraphyletic)	(paraphyletic)	0.94/76	0.94/62
Peridiniales	(paraphyletic)	(paraphyletic)	(paraphyletic)	(paraphyletic)
*Heterocapsa*	1.00/99	1.00/100	1.00/99	–
Suessiales	1.00/90	1.00/93	1.00/94	1.00/100
Dinophysiales	1.00/99	1.00/96	1.00/96	1.00/100
Gonyaulacales	1.00/−	1.00/72	1.00/99	1.00/91
Dinophysiales, Gonyaulacales and Suessiales	1.00/−	1.00/−	1.00/56	–

The first “core” dinoflagellate order to diverge was the athecate Gymnodiniales (Fig. 1, 2, 3, 4), branching paraphyletic in the largest dataset (Fig. 3), being divided into five sub-clades. The first sub-clade to diverge from the Gymnodiniales, and the most basal “core” dinoflagellate, was the highly supported genus *Amphidinium* (Fig. 1, 2, 3, 4; Table 3). *Amphidinium* placed as the sister group to the high to fully supported genus *Gyrodinium* in all but the rDNA inference, where they formed a monophyletic relationship (Fig. 1, 2, 3; Table 3). The low to fully supported family Kareniaceae [17] was next to diverge in all but the rDNA inference, where it alternatively placed terminal to the genus *Gymnodinium sensu stricto* [17]. The three previous Gymnodiniales clades were basal to *Gymnodinium sensu stricto* (Fig. 2–3), excluded with moderate support (1.00/60) in the rDNA+mitochondrial+nuclear protein dataset (Fig. 3). The *Gymnodinium sensu stricto* and several other very closely related genera formed a high to fully supported clade for all datasets (Fig. 1, 2, 3, 4; Table 3). The position of the genus *Akashiwo* was unstable, placing within the Peridiniales with few inferred characters (Fig. 1–2). Increasing character number resulted in a position within the Gymnodiniales (Fig. 3–4). The Noctilucales showed an affinity to *Akashiwo*, and thus Gymnodiniales in the largest dataset (Fig. 3), placing as the sister lineage in all but the rDNA inference, where it alternatively was positioned as the most basal “core” dinoflagellate (Fig. 1).

The basal athecate lineages (Gymnodiniales, Noctilucales, Oxyrrhinaceae and Syndiniales) were excluded from the monophyletic thecate (Dinophysiales, Gonyaulacales, Peridiniales, Prorocentrales and Suessiales) with low support (Fig. 3–4; Table 3). This division was found only in the dataset with the most characters (eight genes). With fewer inferred characters, the position of *Akashiwo* within the Peridiniales resulted in Gymnodiniales being recovered as a polyphyletic order (Fig. 1–2). Additionally, the support for the thecate/athecate split was reduced when a narrower taxon sample was inferred (Fig. 3–4; Table 3). Within the thecate dinoflagellates, the Peridiniales and Prorocentrales were recovered as an unsupported clade (Fig. 3–4), though this was excluded from the Dinophysiales, Gonyaulacales and Suessiales clade with low support in the largest dataset (Fig. 3: Table 3). The Prorocentrales was recovered as a monophyly with low to moderate support when inferred including the mitochondrial genes (Fig. 3–4; Table 3). The *incerta sedis* genus, *Adenoides* [62], which placed as the sister lineage to Prorocentrales, was monophyletic to the prorocentroid clade with the exclusion of mitochondrial cytochrome genes (Fig. 1–2). The high to fully supported genus *Heterocapsa* (Fig. 1, 2, 3; Table 3) was consistently recovered with a placement directly basal to the main Peridiniales clade in all phylogenies excluding the cytochrome genes for this lineage (Fig. 1, 2, 3, 4). The inclusion of the mitochondrial genes resulted in *Heterocapsa* being recovered as the most basal “core” dinoflagellate (Fig. S1). *Heterocapsa* and *Adenoides* were constantly recovered paraphyletic to main Peridiniales clade (Fig. 1, 2, 3, 4). Though, *Azadinium* (Fig. 1) and *Kryptoperidinium* were also recovered paraphyletic to the main Peridiniales monophyly.

The monophyly of Dinophysiales, Gonyaulacales and Suessiales were recovered with broad taxon sampling, with the largest dataset adding support to this relationship (Fig. 1, 2, 3; Table 3). When the taxon sample was reduced, the Suessiales was excluded from this monophyly, alternatively placing as the most basal thecate order, though the placement was unsupported (Fig. 4). The branching pattern for the Dinophysiales, Gonyaulacales and Suessiales clade is uncertain, with no support for a relationship. However, the Dinophysiales was recovered as the unsupported sister clade to the Gonyaulacales when inferring using all eight genes (Fig. 3–4). The Suessiales were monophyletic with high to full support for all datasets, with a more resolved internal branching pattern when inferring more genes (Fig. 1, 2, 3, 4; Table 3). The Dinophysiales, likewise, receive high to full support for their monophyly, with a resolved internal branching pattern for all datasets (Fig. 1, 2, 3, 4; Table 3). The monophyletic Gonyaulacales received low to high support with the addition of inferred characters, though the internal branching pattern was not fully resolved (Fig. 1, 2, 3, 4; Table 3).

The distance-based comparative rate test measured sequence divergence between the different *Heterocapsa* genes (Fig. S3). *Heterocapsa* was compared to the “core” dinoflagellate mean, so if genes were homogenous the distance ratio would be approximately one. Values greater than one indicate a more divergent gene, with values below one indicating a less divergent gene. The distance ratios indicated that *Heterocapsa* rDNA (18S+5.8S+28S) and nuclear protein gene (actin+beta-tubulin+*hsp90*) divergence was less than that of the “core” dinoflagellate mean (1.0), with a 50^th^ percentile range of 0.14360–0.73360, and a 25^th^–75^th^ percentile range of 0.04607–0.77480. The nuclear protein genes diverged approximately 2.38 faster than that of the rDNA. In comparison, the mitochondrial genes were approximately 3 times more divergent than the “core” dinoflagellate mean, as well the rDNA and nuclear protein genes for *Heterocapsa,* with a 50^th^ percentile of 3.58400–3.64000 and a 25^th^–75^th^ percentile of 3.41000–3.68100. This equates to a divergence rate for *Heterocapsa* mitochondrial genes approximately 10 times that of their rDNA genes, and 4 times that of their nuclear protein genes.

### *SxtA* Detection

*SxtA1* and *sxtA4* were not detected in 20 species and five orders. This included two additional orders (Peridiniales and Suessiales) to those already reported (Table 1) [56]. The 18S rDNA control was amplified for all tested species as were the *sxtA* (1/4) positive controls.

## Discussion

### An Improved Dinoflagellate Phylogeny

The phylogenies presented here, with the broadest taxon sampling and largest number of inferred phylogenetic informative nucleotide positions to date, improves the resolution of dinoflagellate in-group relationships. As in previous molecular phylogenetic studies, the dinoflagellates are recovered as a monophyletic lineage [16], [21], [22], [23], [24], with the inferred outgroup taxa. We add statistical support to the phylogenetic backbone allowing us to infer previously unseen relationships and trends between dinoflagellate orders. For the first time, we find that the thecate dinoflagellates have a supported monophyletic origin (Fig. 3–4), diverging from an athecate ancestor. The increased resolution is mostly congruent with the subdivision of orders based on morphological characters, thus broadly supporting the classification based on plate tabulation patterns [6], [94]. Despite this, resolution for some nodes, for example *Akashiwo sanguinea*, can be improved yet further.

This result highlights the importance of using broad taxon sampling, whilst in parallel increasing character number, to further resolve dinoflagellate evolutionary relationships [95], [96], [97]. Previously, “missing characters” has been used as a criterion for taxa exclusion from alignments [16]. In agreement with Wiens (2006), we find little evidence to support the exclusion of taxa based on missing data [81]. Further, we find that the exclusion of such taxa negatively impacts topological resolution, as well as statistical support; with a reduced taxon sample resulting in a more divergent placement of Suessiales, reduced support for the thecate/athecate split, and the recovery of *Kryptoperidinium* external to main Peridiniales clade (Fig. 1, 2, 3, 4; Table 3).

Previous phylogenies inferred with the mitochondrial cytochrome genes *cob* and *cox1*, found *Heterocapsa* to be the most basal “core” dinoflagellate [23], [90]. We also found *Heterocapsa* to be in a basal position when inferring the phylogeny based on mitochondrial genes (Fig. S1). However, the exclusion of *cob* and *cox1* for *Heterocapsa* resulted in a position congruent with both morphological data and phylogenies without mitochondrial genes [16], [30]. The basal position of *Heterocapsa* recovered with mitochondrial genes has been hypothesized as a possible artifact, a result of a faster mutation rate [23]. Indeed, the *cob* and *cox1* sequence for species of *Heterocapsa* were found to be highly diverged compared to that of all other dinoflagellates with a divergence rate approximately 3 times higher, and 4 times that of its own nuclear protein genes. This may result in this lineage being repelled from the in-group and, in contrast, artificially attracted to the out-group. The mitochondrial genes are promising markers for interpreting dinoflagellate evolutionary history, inferring the monophyly of Prorocentrales, improving resolution and increasing support [23]. Thus the exclusion of these markers for all taxa seems to directly oppose the goal of inferring a more resolved phylogeny of the dinoflagellates. Similarly, the exclusion of *Heterocapsa* from inferences would also reduce resolution. Accordingly, and until either the evolution of mitochondria in *Heterocapsa* is fully understood, or enough genetic markers are available to dilute this incongruent signal supported by the trend of mRNA editing of these genes [90], the exclusion of these markers solely for this lineage is warranted. Subsequent exclusion results in a general increase in resolution and support (Fig. 3–4).

The most basal dinoflagellate lineage could not be inferred with certainty from this analysis, with support for the branching pattern between Oxyrrhinaceae and Syndiniales being somewhat inconclusive. A recent study of dinoflagellates based on multiple morphological characters found that the Oxyrrhinaceae was basal to the Syndiniales, which is in line with the results of our largest dataset (Fig. 3) [9]. This is the first phylogeny to conclusively show the sister relationship of these orders, when inferred with broad dinoflagellate taxon sampling [91]. Oxyrrhinaceae and Syndiniales have been excluded from the “core” dinoflagellates, as both lack a dinokaryon [36], [98]. However, previous dinoflagellate phylogenies have lacked either Oxyrrhinaceae [5], [30] or Syndiniales [16], [23], with a highly derived position for *Oxyrrhis* when inferred together [36]. The ancestral position of Oxyrrhinaceae and Syndiniales, both lacking theca, support an athecate dinoflagellate ancestor [5].

In this study, the Gymnodiniales was found to be the most basal “core” dinoflagellate order. Gymnodiniales could be hypothesized to be the sister group to Oxyrrhinaceae and Syndiniales, as the small alveoli are homologous between these orders [5]. However, previous phylogenies have shown the order to be polyphyletic [5], [16], [23], [30]. In contrast, we found the order to show a paraphyletic branching pattern when inferred based on a larger number of genes (Fig. 3–4). This result indicates that it is unlikely that athecate lineages have had thecate ancestors [5], [36], [94], [99]. Similar to previous phylogenies, a polyphyly was observed when the phylogeny was inferred based on fewer characters, as *Akashiwo* showed an affinity to the Peridiniales (Fig. 1–2).

The genus *Amphidinium* was consistently recovered as the most basal “core” dinoflagellate, in all but the phylogeny based on rDNA (Fig. 1), where it shared this position with *Gyrodinium*. This position concurs with previous studies [23], [100]. Unlike the finding for *Heterocapsa*, our results showed no inconsistencies between the mitochondrial and nuclear protein gene phylogenetic signals in *Amphidinium* [23].

The placement of the Noctilucales has long been questioned [5], [16], [36]. The lack of a dinokaryon during particular life cycle stages, would suggest that it may be a basal lineage [6], [10], [11], [12]. However, several novel morphological features and incongruent phylogenies contradict this interpretation [5], [36]. *Noctiluca* was excluded from the presented phylogenies, and instead its most “probable” position inferred from parallel Bayesian analyses. This was done to increase resolution for all taxa, as the cryptic and inconsistent placement of *Noctiluca* reduced phylogenetic support overall. For the bootstrap pseudoreplicates, the position of *Noctiluca* changed from that of a pre-dinoflagellate lineage to having multiple positions within Gymnodiniales, Peridiniales and Suessiales. Only the Bayesian inference of the largest dataset found *Noctiluca* to be the sister to *Akashiwo* with full support. Thus all inferred positions for this taxon are tentative. Similar to previous phylogenies based on rDNA genes [5], [24], [29], [30], we found Noctilucales to be a basal “core” dinoflagellate (Fig. 1). However, increasing the number of inferred characters resulted in an affinity of Noctilucales to *Akashiwo*, and thus the Gymnodiniales (Fig. 3–4). An association between the Noctilucales and the Gymnodiniales has been shown previously with multiple genetic markers [16].

This is the first phylogeny to show support for the monophyly of thecate dinoflagellate orders (Dinophysiales, Gonyaulacales, Peridiniales, Prorocentrales and Suessiales) and has a direct consequence for the evolutionary origin of thecal plates, implying that this morphological trait arose from a single event. Our results are in contrast to previous phylogenies suggesting that these orders are either poly- or paraphyletic, indicating that the thecal characteristic had evolved, or been lost, repeatedly within the “core” dinoflagellates [5], [16], [23]. Taylor (2004), using morphological evidence, hypothesized that thecate dinoflagellates may have arisen from athecate ancestors [36]. The plate increase and plate fragmentation hypotheses did not explain the observed trend [33], [94], [101], [102]. However, aspects of the plate reduction model were seemingly supported, for example the basal position of Gymnodiniales [33], [103]. Further, the plate reduction hypothesis proposed that the Suessiales, with their numerous distinct latitudinal plates, were the most basal thecate dinoflagellate clade, with the Peridiniales giving rise to the Dinophysiales and the Prorocentrales [36]. In general, we did not find support for the Peridiniales being basal to the Dinophysiales and the Prorocentrales, however support was not conclusive (Fig. 2). Interestingly, and in agreement with this hypothesis, we found that the Suessiales was the most basal thecate lineage, with the removal of taxa only possessing rDNA signal (Fig. 4). In contrast to the position for Suessiales observed in Fig. 3, the basal placement is unsupported. The result shows no support for a trend toward sutural loss [36]. Both more genes and more taxa appear to be necessary in order to fully investigate the pattern of thecal plate evolution.

As has been found previously [23], [104], the monophyly of Prorocentrales was only recovered after the inclusion of the mitochondrial cytochrome genes *cob* and *cox1* (Fig. 2). The support for this monophyly was minimal, with *Adenoides,* either placing within the prorocentroid clade (Fig. 1–2), or as its sister group (Fig. 3–4). *Adenoides* has been tentatively placed within Gonyaulacales based on morphological data [105]. However, phylogenetic data (Fig. 3–4) supports the exclusion of this *incertae sedis* genus from the Gonyaulacales, alternatively suggesting an affinity to either the Peridiniales or the Prorocentrales [5], [23], [24]. The Peridiniales has been previously recovered as a polyphyletic lineage [5], [16], [29]. Nevertheless, the result suggests *Prorocentrum* is a derived taxon linked to the peridinioids [36].

The monophyly of Dinophysiales, Gonyaulacales and Suessiales were constantly recovered, with support added for the largest dataset (Fig. 1, 2, 3). This relationship has only been recently seen, albeit without support, with the inference of a large taxon sample for a concatenated 18S and 28S rDNA phylogeny [30]. Previously, the Gonyaulacales has either formed an unsupported monophyly with the Dinophysiales [24] or the Suessiales [5], [16], though alignments analyzed in previous studies generally did not include broad taxon sampling. The Suessiales was recovered as a supported monophyletic lineage with a resolved internal branching pattern [5], [30], [32], [106]. However, the Suessiales was never recovered as a basal order, in contrast to its position based on mitochondrial genes in Zhang et al. (2007) [23]. This, combined with the position of *Heterocapsa* within the Peridiniales, found in this study, rather than as a basal dinoflagellate lineage, as it has been found based purely on mitochondrial genes, suggests that the level of cytochrome mRNA editing is a poor character for determining the most basal lineages of dinoflagellates [90]. The Dinophysiales was found to be a fully supported order, with established internal relationships [5], [30], [107]. The Gonyaulacales was found to be a monophyletic lineage, similar to previous studies [5], [24], [29], [30]. However, the increase in inferred characters added support to this monophyly, increasing resolution for the internal branching pattern.

### The Origin of Saxitoxin

The cyanobacterial STX-pathway is thought to have arisen at least 2100 million years ago [55]. The toxins are seemingly synthesized by similar processes in both cyanobacteria and dinoflagellates [49]. The genes responsible for STX- synthesis have been reported in numerous cyanobacterial species [50], [51], [52], [53], [54]. *SxtA*, the unique starting gene of STX synthesis, has been recently identified in the dinoflagellates [56]. A HGT event between an ancestral STX-producing bacterium and the dinoflagellates has been proposed [56]. This probably occurred before *Alexandrium* and *Pyrodinium* diverged within the order Gonyaulacales. Thus STX-synthesis may have been secondarily lost for some descendent species. *Gymnodinium catenatum,* possessing an *sxtA* sequence that branches within the *Alexandrium* clade, probably independently acquired STX from a later dinoflagellate-dinoflagellate transfer [56].

The results presented here appear to lend some support to this hypothesis; *sxtA* was undetected in any Gonyaulacoids directly basal to the moderately supported clade harboring *Alexandrium* and *Pyrodinium* (Fig. 3). This seems to suggest, in addition to the previous hypothesis, that any proposed HGT event occurred in a recent ancestor of *Alexandrium* and *Pyrodinium* and not deeper within the Gonyaulacales. *SxtA* was not detected in *Ceratium*. Though this genus was found to be basal to the *Alexandrium* and *Pyrodinium* split in the largest dataset, its exclusion was only moderately supported (Fig. 3). In comparison, it was found to be the basal sister to *Alexandrium* in both the rDNA and rDNA+nuclear protein datasets with high support (Fig. 1–2). If a HGT, as proposed, occurred prior to the split of *Alexandrium* and *Pyrodinium*, we may expect *Ceratium* to still possess *sxtA*. A negative result for this genus may therefore, in congruence with the hypothesis, support a secondary loss of STX-synthesis with *sxtA* for some descendent species, additionally including *Coolia*, *Gambierdiscus* and *Pyrocystis* (Fig. 2). However, it may suggest that a HGT event occurred in either *Alexandrium* or *Pyrodinium*, with one of these genera later acquiring STX via a secondary dinoflagellate-dinoflagellate transfer event. This would in-turn reject any theory of secondary loss for *Coolia*, *Gambierdiscus* and *Pyrocystis*. For species of the genus *Alexandrium,* multiple instances of secondary loss can explain the phylogenetic pattern of STX evolution within the genus [69]. To further understand this pattern of loss, a more resolved phylogeny of the order Gonyaulacales is vital. The *sxtA* sequence of *P. bahamense* is currently unavailable, thus sequence comparison remains to be conducted in the future [108].

We were unable to detect *sxtA* for any species external to Gonyaulacales and Gymnodiniales. In addition, *sxtA* was undetected for other species of *Gymnodinium sensu stricto* tested. The dissimilar morphology of Gymnodiniales and Gonyaulacales would support a distant relationship, and phylogenetic studies have tended to support this [16], [24], [36], The current study adds statistical support to this relationship, suggesting that the acquisition of *sxtA* in *Gymnodinium catenatum* was possibly due to a secondary dinoflagellate-dinoflagellate HGT. S*xtA* was not detected in any additional dinoflagellate species and orders to those already reported (Fig. 3–4; Table 1) [56]. The result further demonstrates the capabilities of the *sxtA* primers for the detection of environmental STX [56].

### Future Aims and Perspectives

This study improves dinoflagellate in-group resolution considerably, however some relationships remain unclear. Presently, the Marine Microbial Eukaryotic Transcriptome Project (https://www.marinemicroeukaryotes.org/) are sequencing 142 dinoflagellate strains, spanning eight orders. Once these data become publicly available, it will be possible to further increase the phylogenetic resolution of dinoflagellates. To increase resolution yet further, a focus is needed on *incerta sedis* taxa. These species are either heterotrophic and unculturable, or rare and not available in culture collections. For example the benthic genera *Rhinodinium* [109], *Cabra* [110], *Pseudothecadinium* [111] and *Halostylodinium* [112] have unclear family level affinities. *Adenoides* [105], *Plagiodinium* [113], *Pileidinium* [114] and *Tovelliaceae* [115] have unclear order-level affinities. This study demonstrates that improved taxon sampling is as important, if not more important, as increasing the number of inferred genes [95], [96], [97] in order to obtain a resolved phylogeny.

In relation to STX, the characterization of additional pathway genes is vital to determine the toxin evolution. This is needed to further corroborate the HGT theory and determine where in dinoflagellate evolution this may have occurred. It is important to understand the pattern of STX loss further. For example, which genes have been lost and from what lineages? Have genes been retained and are they being transcribed? Are there remnants of *sxtA* in the genome of non-toxic species? Such questions highlight the importance of work on non-STX producing species, whilst most focus has been on their toxic sisters.

## Supporting Information

Figure S1 Phylogenetic tree of dinoflagellates inferred from rDNA, mitochondrial and nuclear protein genes. Concatenated phylogeny, inferred from 18S+5.8S+28S+*cob*+*cox1*+actin+beta-tubulin+*hsp90* (7138 characters). The tree is inferred as in Figure. 3, however, *Heterocapsa* cytochrome has not been excluded. The tree is reconstructed with Bayesian inference (MrBayes). Numbers on the internal nodes represent posterior probability and bootstrap values (>50%) for MrBayes and RAxML (ordered; MrBayes/RAxML). Black circles indicate a posterior probability value of 1.00 and bootstrap >90%. *N. scintilans* is represented with a dashed branch as this taxon was excluded from the inference; alternatively its most “probable” placement was determined from a parallel Bayesian analysis. * Denotes taxa sequences generated from this study. See Table S2 for a full listing of accessions used.
(EPS)

Figure S2 Phylogenetic tree of dinoflagellates inferred from mitochondrial and nuclear protein genes. Concatenated phylogeny, inferred from *cob*+*cox1*+actin+beta-tubulin+*hsp90* (4238 characters). The cytochrome genes *cob* and *cox1* for *H. triquetra* were excluded from the inference. The tree is reconstructed with ML (RAxML). Numbers on the internal nodes represent bootstrap values (>50%). * Denotes taxa sequences generated from this study. See Table S2 for a full listing of accessions used. The observed topology demonstrates good topological congruence with the equivalent rDNA inference: *l_cong_ P*-value <0.05.
(EPS)

Figure S3 Comparative rate test of *Heterocapsa* genes. The distance ratio of *Heterocapsa* to nine “core” dinoflagellate (ingroup) taxa compared to mean pairwise distance for the same ingroup taxa, calculated for every gene. When the ratio is greater than one, *Heterocapsa* can be considered more divergent than the “core” dinoflagellate mean. A ratio less than one, the “core” dinoflagellate mean is more divergent than *Heterocapsa*. When the ratio is approximately one divergence between *Heterocapsa* and the “core” dinoflagellate mean is homogenous. The box spans the 25^th^ to 75^th^ percentile with the horizontal bar indicating the 50^th^ percentile. The whiskers include the entire range from 0 to 100 percentile.
(EPS)

Figure S4 18S rDNA phylogeny (1724 characters): The tree is reconstructed with ML (RAxML). Numbers on the internal nodes represent bootstrap values (>50%). * Denotes taxa sequences generated from this study. Accessions for each taxon are shown in brackets.
(EPS)

Figure S5 5.8S rDNA phylogeny (151 characters): The tree is reconstructed with ML (RAxML). Numbers on the internal nodes represent bootstrap values (>50%). * Denotes taxa sequences generated from this study. Accessions for each taxon are shown in brackets.
(EPS)

Figure S6 28S rDNA phylogeny (1025 characters): The tree is reconstructed with ML (RAxML). Numbers on the internal nodes represent bootstrap values (>50%). * Denotes taxa sequences generated from this study. Accessions for each taxon are shown in brackets.
(EPS)

Figure S7 Actin mRNA phylogeny (752 characters): The tree is reconstructed with ML (RAxML). Numbers on the internal nodes represent bootstrap values (>50%). * Denotes taxa sequences generated from this study. Accessions for each taxon are shown in brackets.
(EPS)

Figure S8 Beta tubulin mRNA phylogeny (842 characters): The tree is reconstructed with ML (RAxML). Numbers on the internal nodes represent bootstrap values (>50%). * Denotes taxa sequences generated from this study. Accessions for each taxon are shown in brackets.
(EPS)

Figure S9 Cytochrome Oxidase 1 mRNA phylogeny (892 characters): The tree is reconstructed with ML (RAxML). Numbers on the internal nodes represent bootstrap values (>50%). * Denotes taxa sequences generated from this study. Accessions for each taxon are shown in brackets.
(EPS)

Figure S10 Cytochrome B mRNA phylogeny (620 characters): The tree is reconstructed with ML (RAxML). Numbers on the internal nodes represent bootstrap values (>50%). * Denotes taxa sequences generated from this study. Accessions for each taxon are shown in brackets.
(EPS)

Figure S11 Heat shock protein 90 gDNA phylogeny (1132 characters): The tree is reconstructed with ML (RAxML). Numbers on the internal nodes represent bootstrap values (>50%). * Denotes taxa sequences generated from this study. Accessions for each taxon are shown in brackets.
(EPS)

Table S1 Primers specifically designed for this study or used in previous studies. T_M_ calculated using OligoCalc [73]. Annealing site is an approximation and can vary slightly between species. The primer-pairs and PCR annealing temperature used were as follows. 52°C: SL+ DinoActinR2, DinoActinF2+AUAP, SL+ DinoBtubR1, SL+ DinoBtubR2, NSF83+1528R, 18F1574+28R691new, 18F1574+28R691new, 28F341+28R1318. 54°C: DinoActinF1+AUAP, DinoBtubF1+AUAP, DinoBtubF2+AUAP, Btub305F+AUAP, SL+Btub305R, DinoCYTbF1+AUAP, DinoCYTbF2+AUAP, DinoCOXF2+AUAP, DinoRhsp90F2+DinoRhsp90R2**.** 56°C: CYTB343F+AUAP, COX211F+ COX1021R, COX631F+AUAP, COX631F+ COX1021R, 18SF8+ ITSR01. 57°C: Actin943F+AUAP. 64°C: Sxt001+Sxt002, Sxt007+Sxt008.
(DOC)

Table S2 Accession numbers of the species represented in the supermatrices. Accessions amplified from this study are highlighted with an asterisk.
(DOC)
