# Supplementary material for: When Naked Became Armored: An Eight-Gene Phylogeny Reveals Monophyletic Origin of Theca in Dinoflagellates
Source: PLoS One. 2012 Nov 19;7(11):e50004. doi: 10.1371/journal.pone.0050004 (PMC3501488; doi:10.1371/journal.pone.0050004)
Supplement: Table S1 — Primers specifically designed for this study or used in previous studies. TM calculated using OligoCalc [73]. Annealing site is an approximation and can vary slightly between species. The primer-pairs and PCR annealing temperature used were as follows. 52°C: SL+ DinoActinR2, DinoActinF2+AUAP, SL+ DinoBtubR1, SL+ DinoBtubR2, NSF83+1528R, 18F1574+28R691new, 18F1574+28R691new, 28F341+28R1318. 54°C: DinoActinF1+AUAP, DinoBtubF1+AUAP, DinoBtubF2+AUAP, Btub305F+AUAP, SL+Btub305R, DinoCYTbF1+AUAP, DinoCYTbF2+AUAP, DinoCOXF2+AUAP, DinoRhsp90F2+DinoRhsp90R2. 56°C: CYTB343F+AUAP, COX211F+ COX1021R, COX631F+AUAP, COX631F+ COX1021R, 18SF8+ ITSR01. 57°C: Actin943F+AUAP. 64°C: Sxt001+Sxt002, Sxt007+Sxt008. (DOC) [file pone.0050004.s012.doc]

| **Primer name** | **Primer direction** | **Primer sequence 5’-3’** | **Annealing site 5’-3’** | **T_M_** | **Reference** |
| --- | --- | --- | --- | --- | --- |
| DinoActinF1 | F | GAYGARGCDCAGAGCAAGC | 169-187 | 59.5 | This study |
| DinoActinF2 | F | ATCATGGTSGGCATGGAC | 130-147 | 56.0 | This study |
| DinoActinR2 | R | TTGGAGATCCACATCTGCTG | 1060-1079 | 57.3 | This study |
| Actin943F | F | ATGAAGATCAAGGTNGTNGC | 976-995 | 55.3 | This study |
| DinoBtubF1 | F | GGHGCNAARTTYTGGGAGG | 49-67 | 58.5 | This study |
| DinoBtubF2 | F | GDGCMAAGTTCTGGGARGT | 50-68 | 57.4 | This study |
| DinoBtubR1 | R | AGGTGGTTCAGGTCHCCGTA | 665-683 | 60.0 | This study |
| DinoBtubR2 | R | YTCWCCDGTGTACCARTGCAA | 1183-1203 | 58.5 | This study |
| Btub305F | F | TSCAGGGBTTCCAGATGT | 389-406 | 55.2 | This study |
| Btub305R | R | ACATCTGGAAVCCCTGSA | 389-406 | 55.2 | This study |
| DinoCYTbF1 | F | WCHGGWATCTTCTTAGCTTTACATTA | 73-98 | 57.4 | This study |
| DinoCYTbF2 | F | TTRTCACWGGAATCTTMTTAGSTTT | 68-92 | 56.4 | This study |
| CYTB343F | F | GGACAAATGAGTTTMTGGGG | 343-362 | 56.3 | This study |
| DinoCOXF2 | F | CCATTAAGCACKTCTTTYMTGAGTT | 349-373 | 58.9 | This study |
| COX211F | F | ATCTTTCAAGGRTCTCCWGAAGTG | 211-234 | 60.2 | This study |
| COX631F | F | TTTGGAGGAGATCCTRTWCTCTAT | 631-654 | 58.4 | This study |
| COX1021R | R | CCAAGAATTACTCCTGTTGASCC | 1021-1043 | 60.6 | This study |
| DinoRhsp90F2 | F | ATCCGSTAYGAGTCVATCAC | 46-65 | 57.6 | This study |
| DinoRhsp90R2 | R | ACCTTGTCKCCSARVACCT | 1577-1595 | 58.1 | This study |
| SL | F | DCCGTAGCCATTTTGGCTCAAG | Variable | 57.0 |  |
| AUAP | R | GGCCACGCGTCGACTAGTAC | Variable | 57.9 | Invitrogen |
| NSF83 | F | GAAACTGCGAATGGCTCATT | 82-101 | 49.7 |  |
| 1528R | R | TGATCCTTCTGCAGGTTCACCTAC | 1777-1800 | 57.4 |  |
| 18SF8 | F | TTGATCCTGCCAGTAGTCATATGCTTG | 8-34 | 58.2 |  |
| ITSR01 | R | CCTTGTTACGACTTCTCCTTCCTC | 1748-1771 | 57.4 |  |
| 18F1574 | F | CCCGTCGCTCCWACYGATT | 1645-1663 | 55.4 |  |
| 28R691new | R | CTTGGWCCGTGTTTCAAGAC | 3020-3039 | 51.8 |  |
| 28F341 | F | AGCACACAAGTACCATGAGG | 2634-2653 | 51.8 |  |
| 28R1318 | R | TCGGCAGGTGACTTGTTACACAC | 3627-3649 | 57.1 |  |
| M13F | F | GTAAAACGACGGCCAG | - | 45.9 | Invitrogen |
| M13R | R | CAGGAAACAGCTATGA | - | 40.8 | Invitrogen |
| Sxt001 | F | TGCAGCGMTGCTACTCCTACTAC | 904-926 | 57.1 |  |
| Sxt002 | R | GGTCGTGGTCYAGGAAGGAG | 1429-1449 | 55.9 |  |
| Sxt007 | F | ATGCTCAACATGGGAGTCATCC | 3174-3195 | 54.8 |  |
| Sxt008 | R | GGGTCCAGTAGATGTTGACGATG | 3865-3888 | 57.1 |  |
