# Supplementary material for: When Naked Became Armored: An Eight-Gene Phylogeny Reveals Monophyletic Origin of Theca in Dinoflagellates
Source: PLoS One. 2012 Nov 19;7(11):e50004. doi: 10.1371/journal.pone.0050004 (PMC3501488; doi:10.1371/journal.pone.0050004)
Supplement: Table S2 — Accession numbers of the species represented in the supermatrices. Accessions amplified from this study are highlighted with an asterisk. (DOC) [file pone.0050004.s013.doc]

| **Species/Taxon** | **18S** | **5.8S** | **28S** | **Actin (mRNA)** | **Beta Tubulin (mRNA)** | **Cytochrome Oxidase 1 (mRNA)** | **Cytochrome B (mRNA)** | ***Hsp90* (genomic)** |
| --- | --- | --- | --- | --- | --- | --- | --- | --- |
| **Dinophysiales** |  |  |  |  |  |  |  |  |
| *Amphisolenia bidentata* | GU196149 |  | FJ808682 |  |  |  |  |  |
| *Dinophysis accuminata* | AJ506972 | AM931580 | AY277640 | HQ391384 |  | EU130565 | EU130567 |  |
| *Dinophysis caudata* | EU780644 | EU780644 | EU780644 | HQ391397 |  |  |  |  |
| *Ornithocercus magnificus* | EU780649 | EU780649 | EU780649 |  |  |  |  |  |
| *Ornithocercus quadratus* | EU780647 | EU780647 | EU780647 |  |  |  |  |  |
| *Phalacroma* cf. *rotundatum* | EU780657 | EU780657 | EU780657 |  |  |  |  |  |
| *Phalacroma rapa* | EU780655 | EU780655 | EU780655 |  |  |  |  |  |
| **Gonyaulacales** |  |  |  |  |  |  |  |  |
| *Alexandrium affine* | AJ535375 | AJ632095 | AY831409 |  |  | EF377324 | EF036543 |  |
| *Alexandrium fundyense* | JF521624 | JF521624 | JF521624 | *JX262500 | *JX262511 | *JX262520 | *JX262530 | *JX262540 |
| *Alexandrium minutum* | JF521634 | JF521634 | JF521634 | *JX262501 | *JX262512 | *JX262521 | *JX262531 | *JX262541 |
| *Amylax triacantha* | AB375869 |  | EF613350 |  |  |  |  |  |
| *Ceratium furca* | AJ276699 | AJ276700 | AY027908 |  |  |  |  |  |
| *Ceratium longipes* | DQ388462 | EU927566 | EU165305 |  |  | EF036570 | EF036546 |  |
| *Ceratocorys horrida* | DQ388456 | EU927577 |  |  |  | EF036571 | EF036547 |  |
| *Coolia canariensis* | HQ897282 |  | HQ897278 |  |  |  |  |  |
| *Coolia monotis* | EF492487 |  | CMU92258 |  |  | EF036572 |  |  |
| *Fragilidium subglobosum* | AF033869 |  | AF260387 |  |  |  |  |  |
| *Gambierdiscus australes* | EF202895 | *JX262494 | EF202972 |  |  |  |  |  |
| *Gambierdiscus toxicus* | EF202883 |  | EF202961 |  |  | EF036575 | EF036550 |  |
| *Gonyaulax cochlea* | AF274258 |  | FJ93957 |  |  | EF036576 | EF036551 |  |
| *Gonyaulax spinifera* | AF022155 | AF051832 | AY154960 |  |  |  |  |  |
| *Lingulodinium polyedrum* | AF274269 | FJ823577 | EF613357 | AY423582 | BP743312 | CD810287  CD809863  CD810813  BP743044 | BP742299  CD810481  CD810020 |  |
| *Protoceratium reticulatum* | AY421790 | EU532486 | AF260386 |  |  | EF036589 | EF036560 |  |
| *Pyrocystis lunula* | AF274274 |  | BQ280395 |  |  | EF036590 | EF036561 |  |
| *Pyrocystis noctiluca* | AF022156 |  | FJ939576 |  |  | EF036591 | EF036562 |  |
| *Pyrodinium bahamense* | DQ500120 | AF145225 | AY154959 |  |  |  |  |  |
| *Thecadinium kofoidii* | GU295204 | *JX262497 | GU295207 | *JX262509 | *JX262519 | *JX262529 |  | GU295215 |
| **Gymnodiniales** |  |  |  |  |  |  |  |  |
| *Akashiwo sanguinea* | AY831412 | AY831412 | AY831412 | EF640329 |  | EF036566 | EF036542 | GU295192 |
| *Amphidinium carterae* | EU046334 | FJ823528 | AY460586 | EU742722 | CF066994  CF065002  CF067402 | EF036568 | EF036544 | EU876701 |
| *Amphidinium mootonorum* | GU295202 |  | AY455676 |  |  | *JX262523 | *JX262532 | GU295199 |
| *Amphidinium_massartii* | AF274255 | EU927576 | AY455670 | *JX262502 |  | *JX262522 | *JX262533 | *JX262542 |
| *Brachidinium capitatum* | HM066998 | HM066999 | HM067000 |  |  |  |  |  |
| *Dissodinium pseudolunula* | FJ473378 |  | AY526523 |  |  |  |  |  |
| *Gymnodinium aureolum* | AY999082 | AM184203 | DQ917486 | *JX262504 | *JX262514 | *JX262525 | *JX262535 | *JX262544 |
| *Gymnodinium catenatum* | DQ779990 | AY506592 | DQ779989 | EF640327 |  |  | EF036552 |  |
| *Gymnodinium dorsalisulcum* | DQ837534 |  | DQ336190 |  |  |  |  |  |
| *Gymnodinium fuscum* | AF022194 |  | AF200676 |  |  |  |  | GU295194 |
| *Gyrodiniellum shiwhaense* | FR720082 | FR720082 | FR720082 |  |  |  |  |  |
| *Gyrodinium dominans* | FN669510 | FN669510 | AY571370 |  |  |  |  |  |
| *Gyrodinium rubrum* | AB120003 |  | AY571369 |  |  |  |  |  |
| *Gyrodinium spirale* | AB120001 |  | AY571371 |  |  |  |  |  |
| *Karenia brevis* | EF492501 | AF352827 | EU165308 | EX964342  EX969616 | EU078557 | CO059135  EF036580  CO062170 | EF036555  FK855467 | AM184117 |
| *Karenia mikimotoi* | EF492505 | FJ823564 | EF469238 | EF640326 |  | EF036581 |  | AM184120 |
| *Karlodinium veneficum* | AY245692 | AJ557025 | DQ114466 | DQ867056 | EF134086 | AM773802 | EF443012  EC161023 | AM184121 |
| *Lepidodinium chlorophorum* | AM184122 | *JX262495 | AB367942 | *JX262505 | *JX262515 | *JX262526 | *JX262536 | AM184119 |
| *Lepidodinium viride* | DQ499645 | DQ499645 | DQ499645 |  |  |  |  |  |
| *Nematodinium* sp. BSL-2009a | FJ947039 |  | FJ947041 |  |  |  |  |  |
| *Pheopolykrikos hartmannii* | AY421789 |  | FJ947045 |  |  |  |  |  |
| *Polykrikos kofoidii* | DQ371291 |  | FJ947043 |  |  |  |  |  |
| *Spiniferodinium galeiforme* | GU295203 |  | GU295206 |  |  |  |  | GU295214 |
| *Takayama* cf. *pulchellum* | AY800130 | AY764179 | AY764178 |  |  |  |  |  |
| *Warnowia* sp. BSL-2009a | FJ947040 |  | FJ947042 |  |  |  |  |  |
| **Noctilucales** |  |  |  |  |  |  |  |  |
| *Noctiluca scintillans* | AF022200 | GQ380592 | GQ380592 | EF134225 |  | EF036583 |  | AB297471 |
| **Oxyrrhinaceae** |  |  |  |  |  |  |  |  |
| *Oxyrrhis marina* | AY56641 | AY566418 | EG736066  EG738111 | EG735829  EG734172 | EG738530  EG733090  EG744590 | EF680822 | EF036557 | AY391258 |
| **Peridiniales** |  |  |  |  |  |  |  |  |
| *Adenoides eludens* | EF492484 | *JX262493 | FJ939580 | *JX262499 | *JX262510 | EF036565 | EF036541 | *JX262539 |
| *Azadinium obesum* | GQ914935 | FJ766093 | GQ914936 |  |  |  |  |  |
| *Azadinium spinosum* | *JX262491 | HQ324892 | HQ324896 | *JX262503 | *JX262513 | *JX262524 | *JX262534 | *JX262543 |
| *Crypthecodinium cohnii* | M64245 | FJ823534 | FJ939575 | AF421536 | AF421537 | AF487783 | AF403221 |  |
| *Cryptoperidiniopsis brodyi* | DQ991374 |  | DQ991377 |  |  | EF036585 | EF036558 |  |
| *Duboscquodinium collinii* | HM483399 | HM483399 | HM483399 |  |  |  |  |  |
| *Durinskia baltica* | GU999528 |  | GU999529 |  |  |  |  |  |
| *Ensiculifera* aff. *loeblichii* | HQ845328 | HQ845328 | HQ845328 |  |  |  |  |  |
| *Gloeodinium montanum* | EF058238 |  | EF205003 |  |  |  |  |  |
| *Heterocapsa hallii* | AF033865 |  | AF033867 |  |  |  |  |  |
| *Heterocapsa pygmeae* | AF274266 | FJ823558 | FJ939577 |  |  |  |  |  |
| *Heterocapsa triquetra* | AF022198 | HQ902268 | EF613355 | DT386216  EF640328  DT385734 | DT385728  AF482414  DT384505 | EF036578 | EF036554 | AY729855 |
| *Kryptoperidinium foliaceum* | EF492508 | FJ823570 | EF052684 |  |  |  |  | AY713394 |
| *Luciella masanensis* | EU048553 | EU048553 | EU048553 |  |  |  |  |  |
| *Pentapharsodinium dalei* | *JX262492 | *JX262496 | *JX262498 | *JX262506 | *JX262516 |  |  | *JX262545 |
| *Pentapharsodinium tyrrhenicum* | AF022201 | AY499512 | DQ167859 |  |  |  |  |  |
| *Peridiniopsis niei* | HM596542 | HM596550 | HM596555 |  |  |  |  |  |
| *Peridiniopsis penardii* | AB353771 | HM596554 | HM596558 |  |  |  |  |  |
| *Peridinium cinctum* | EF058245 | FJ823578 | EF205011 |  |  |  |  |  |
| *Peridinium willei* | AF274280 | AB232669 | EF205012 |  |  |  |  | GU295195 |
| *Pfiesteria piscicida* | AY245693 | DQ991382 | AY112746 | EF640323 |  | AF463413 | AF357518 |  |
| *Protoperidinium claudicans* | AB255833 |  | AB255842 |  |  |  |  |  |
| *Protoperidinium minutm* | GQ227501 |  | GQ227502 |  |  |  |  |  |
| *Pseudopfiesteria shumwayae* | AF080098 | AF352345 | AY245694 | EF640324 |  | EF036586 | DQ082988 |  |
| *Scrippsiella sweeneyae* | AF274276 | AY499528 | AY628428 |  |  | EF036593 | EF036563 |  |
| *Scrippsiella trochoidea* | EF492513 | FJ823592 | HM483396 | *JX262508 | *JX262518 | *JX262528 | *JX262538 | GU295213 |
| *Stoeckeria algicida* | AJ841809 | EU978468 | EU978467 |  |  |  |  |  |
| *Thoracosphaera heimii* | AF274278 | AY327463 | EF205018 |  |  |  |  |  |
| *Tintinnophagus acutus* | HM483397 | HM483397 | HM483397 |  |  |  |  |  |
| **Prorocentrales** |  |  |  |  |  |  |  |  |
| *Prorocentrum concavum* | Y16237 |  | EF566751 |  |  |  |  |  |
| *Prorocentrum consutum* | FJ842379 |  | FJ842378 |  |  |  |  |  |
| *Prorocentrum donghaiense* | AY803743 | AY465116 | AY822610 |  |  | EF036587 | DQ336056 |  |
| *Prorocentrum lima* | AB189780 | FJ823582 | DQ336179 | EF640330 |  | EF377325 | EF03655 |  |
| *Prorocentrum micans* | EF492511 | EU244467 | X16108 | EF640331 |  | EF036588 | AY745238 | AY391260 |
| *Prorocentrum minimum* | AY803741 | EU244473 | EU780639 | AF512889 | EF134371 | AF463415 | AY030285 | GU295201 |
| **Suessiales** |  |  |  |  |  |  |  |  |
| *Baldinia anauniensis* | EF052682 |  | EF052683 |  |  |  |  |  |
| *Biecheleria baltica* | EF058252 | DQ167868 | EF205019 |  |  |  |  |  |
| *Pelagodinium beii* | U41087 | DQ198026 | DQ198074 |  | EF432512 |  |  |  |
| *Polarella glacialis* | EF434275 | FJ823580 | AY036081 | *JX262507 | *JX262517 | *JX262527 | *JX262537 | GU295196 |
| *Protodinium simplex* | DQ388466 | AY686651 | EF205014 |  | EF432488 | EF036577 | EF036553 | GU295211 |
| *Symbiodinium microadriaticum* | EF492496 | AF333505 | SMU63483 |  |  | EF036594 | DQ082985 |  |
| *Symbiodinium* sp. C3 | EH037985 | EH057907 | FJ529524 | EH035946  EH058179 | EH057872  EH037669 | FE864081  EH03797 | EH037604  EH037615 |  |
| **Syndiniales** |  |  |  |  |  |  |  |  |
| *Amoebophrya* sp. ex *Akashiwo sanguineaum* | HM483395 | HM483395 | HM483395 |  |  |  |  |  |
| *Amoebophrya* sp. ex *Gymnodinium instriatum* | AF472554 | HM483394 | HM483394 |  |  |  |  |  |
| *Hematodinium* sp. | FJ844431 | JN641990 | JN641990 | HE609031 |  | HE610721 | HE610722 |  |
| **Outgroup** |  |  |  |  |  |  |  |  |
| *Cryptosporidium parvum* | X64341 | AF093014 | AF040725 | XM_001388245 | XM_627803 |  |  |  |
| *Eimeria tenella* | AF026388 | AY779514 | AF026388 | AM948365  AM948366  BM321663 | ETU19268  AM264193 | AB564272 | CD659755 | AF042329 |
| *Perkinsus marinus* | AF497479 | EF204014 | EU919450 | AY436365 | XM_002772788 | HQ670240 | HQ670241 | AY391259 |
| *Theileria parva* | AF013418 |  | U03602 | XM_760378 | XM_759937 | Z23263 |  | XM_759717 |
| *Toxoplasma gondii* | M97703 | L25635 | AF076901 | XM_002369622 | XM_002369950 | AF187882 | AF023246 | XM_002368237 |
| **TOTAL taxa** | **104** | **74** | **103** | **36** | **26** | **47** | **43** | **31** |
| **Alignment length pre G-blocks** | **1906** | **186** | **2167** | **1128** | **1263** | **1358** | **1034** | **1820** |
| **Alignment length post G-blocks** | **1724** | **151** | **1025** | **1128** | **1263** | **1338** | **930** | **1698** |
